# Supplementary material for: Minimal biophysical model of combined antibiotic action
Source: PLoS Comput Biol. 2021 Jan 7;17(1):e1008529. doi: 10.1371/journal.pcbi.1008529 (PMC7817058; doi:10.1371/journal.pcbi.1008529)
Supplement: S1 Appendix — (PDF) [file pcbi.1008529.s001.pdf]

S1 Appendix
for
Minimal biophysical model of combined antibiotic action

Bor Kavčič[1], Gašper Tkačik[1], Tobias Bollenbach[2,3,*]

December 10, 2020

[1]Institute of Science and Technology Austria, Klosterneuburg, Austria
[2]Institute for Biological Physics, University of Cologne, Cologne, Germany
[3]Center for Data and Simulation Science, University of Cologne, Cologne, Germany
*t.bollenbach@uni-koeln.de

1 Parameter reduction and the bifurcation point

In the following we derive expressions that constrain the parameter space in which the system is bistable. We start by rewriting the steady-state solution with new variables, which will facilitate reduction of parameters. We recast Eq (4) so as to express $a_{\mathrm{ex}}/2\mathrm{IC}_{50}^{*}$ as function of growth rate; this is possible since $a_{\mathrm{ex}}/2\mathrm{IC}_{50}^{*}$ occurs linearly in the equation. At this stage we introduce new variables and rewrite parameters in a dimensionless form:

$$a_{\mathrm{ex}} = c \times \mathrm{IC}_{50}, \tag{S1.1a}$$
$$\lambda = y \times \lambda_0, \tag{S1.1b}$$
$$\lambda_0^{*} = \alpha \times \lambda_0, \tag{S1.1c}$$

where IC_{50} is concentration needed to halve the growth rate and λ_0 is a drug-free growth rate. These definitions lead to:

$$y\left[\frac{1}{4}\alpha^2 + \frac{1}{2}\frac{\mathrm{IC}_{50}}{\mathrm{IC}_{50}^{*}}c\alpha\right] = \frac{1}{4}\alpha^2 + y^2 - y^3. \tag{S1.2}$$

To remove the ratio $\mathrm{IC}_{50}/\mathrm{IC}_{50}^{*}$ from the equation, we use the expression from Ref. [1] that states

$$\frac{\mathrm{IC}_{50}}{\mathrm{IC}_{50}^{*}} = \frac{1}{2}\left(\frac{\lambda_0}{\lambda_0^{*}} + \frac{\lambda_0^{*}}{\lambda_0}\right) = \frac{1}{2}\left(\alpha + \frac{1}{\alpha}\right) = \frac{1}{2}\left(\frac{\alpha^2+1}{\alpha}\right). \tag{S1.3}$$

This relation is obtained by plugging $y = 1/2$ and $c = 1$ into Eq (S1.2). Finally, we recast Eq (S1.2) into

$$c = \frac{1}{\alpha^2+1}\left(\frac{\alpha^2}{y} - \alpha^2 + 4y - 4y^2\right). \tag{S1.4}$$

This equation expresses the relative concentration c as a function of relative growth rate y in which α is the sole parameter. Fitting an implicit function can be challenging; we can estimate α from equating the implicitly calculated derivative of Eq (S1.4) to the derivative of the Hill function with steepness parameter n, when both derivatives are evaluated at $c = 1$. An estimate for the response parameter is then $\alpha \approx \sqrt{1/(n-1)}$.

1.1 Bifurcation point

To determine the critical value of the parameter α below which bistable concentration region exists, we consider the stationary points of Eq (S1.4). The derivative reads:

$$\frac{\mathrm{d}c}{\mathrm{d}y} = \frac{1}{\alpha^2+1}\left(-\frac{\alpha^2}{y^2}+4-8y\right). \tag{S1.5}$$

Considering the equation

$$8y^3-4y^2+\alpha^2=0, \tag{S1.6}$$

we can determine values of y where the derivative of Eq (S1.5) is 0. Since the equation is of third order, we expect either three real roots or one real together with a conjugated pair of complex roots. The solutions [found using Mathematica (Wolfram Research, 11.3) function `Solve`] are:

$$y_1 = \frac{1}{6}\left[\sqrt[3]{\frac{3}{2}\alpha\left(\sqrt{81\alpha^2-12}-9\alpha\right)+1}+\frac{1}{\sqrt[3]{(3/2)\alpha\left(\sqrt{81\alpha^2-12}-9\alpha\right)+1}}+1\right], \tag{S1.7a}$$

$$y_2 = \frac{1}{24}\left[2(-2)^{2/3}\sqrt[3]{3\alpha\left(\sqrt{81\alpha^2-12}-9\alpha\right)+2}-\frac{4\sqrt[3]{-2}}{\sqrt[3]{3\alpha\left(\sqrt{81\alpha^2-12}-9\alpha\right)+2}}+4\right], \tag{S1.7b}$$

$$y_3 = \frac{1}{24}\left[-2\sqrt[3]{-1}2^{2/3}\sqrt[3]{3\alpha\left(\sqrt{81\alpha^2-12}-9\alpha\right)+2}+\frac{4(-1)^{2/3}}{\sqrt[3]{(3/2)\alpha\left(\sqrt{81\alpha^2-12}-9\alpha\right)+1}}+4\right]. \tag{S1.7c}$$

We note that whether all roots are real is determined by the sign of $27\alpha^2-4$; this leads to a critical value of $\alpha_{\mathrm{crit}} = 2/(3\sqrt{3}) \approx 0.385$–the system has a bistable region when $\alpha < \alpha_{\mathrm{crit}}$. To obtain the span of concentrations at which two bistable growth rates exist (Fig 1D), we plug Eqs (S1.7a) and (S1.7c) into Eq (S1.4).

1.2 Linearized case of constitutively expressed resistance gene

As noted in the main text, for high values of K_{rem} the Michaelis-Menten equation linearizes. In this case we can reuse the expression for growth-dependent efflux from Ref. [1]. Using the parameter reduction from above, the generic solution is

$$\begin{aligned} y^3 &- y^2\left(1-\frac{1}{4}\Omega\alpha^2\right)+ \\ &+ y\left[\frac{a_{\mathrm{ex}}}{2\mathrm{IC}_{50}^*}\alpha+\frac{1}{4}(1-\Omega)\alpha^2\right]-\frac{1}{4}\alpha^2=0. \end{aligned} \tag{S1.8}$$

where $\Omega = V'_{\mathrm{max}}/(p_{\mathrm{out}}K_{\mathrm{rem}})$. This expression can be rewritten into reduced form as

$$\begin{aligned} c &= \frac{1}{1+\alpha^2(1+\Omega/2)}\times \\ &\times \left[\frac{\alpha^2}{y}-(1-\Omega)\alpha^2+4y\left(1-\frac{\Omega\alpha^2}{4}\right)-4y^2\right]. \end{aligned} \tag{S1.9}$$

Here, we used the relation that follows directly from Eq (S1.8); $\mathrm{IC}_{50}/\mathrm{IC}_{50}^* = (1/2\alpha)\times\left[1+\alpha^2(1+\Omega/2)\right]$. By solving $\mathrm{d}c/\mathrm{d}y = 0$ we obtain a condition $-64\alpha^2+432\alpha^4+48\alpha^4\Omega-12\alpha^6\Omega^2+\alpha^8\Omega^3 \leq 0$ for bistability to exist; heuristically, we note that the leading term in Ω is of the third power and positive and will for high values of Ω make the expression necessarily positive and will thus make the system monostable. This illustrates the importance of non-linearity in the expression describing antibiotic removal [Eq (19)]; the presence of positive feedback alone is not sufficient for the occurrence of bistability. In the limit $\Omega \to \infty$, Eq (S1.9) becomes simply $y = 1-c/2$ for $c \leq 2$ and $y = 0$ otherwise.

2 Calculation of dose-response surface for additive interaction and Loewe interaction score

An additive interaction is characterized by linear isoboles. In this section we briefly sketch the derivation. Let c_A and c_B be relative concentrations (measured in IC_{50} of respective antibiotics) of antibiotics A and B, respectively. Individual dose-response curves are given by f_A and f_B. To construct the additive surface, the growth rate in any point (c_A, c_B) has to be calculated solely from known responses to individual drugs. As isoboles are linear, there is an isobole that passes through this point but terminates in some unique $(c_{A,0}, 0)$ and $(0, c_{B,0})$, from which it follows that $c_B = c_{B,0}(1 - c_A/c_{A,0})$. Since $c_{B,0} = f_B^{-1}(f_A(c_{A,0}))$, the solution of $c_B = f_B^{-1}(f_A(c_{A,0})) \times (1 - c_A/c_{A,0})$ for $c_{A,0}$ is sought, which depends on c_A and c_B. If such solution is termed $\hat{c}_{A,0}(c_A, c_B)$ then the whole dose-response surface is given as $f_A(\hat{c}_{A,0}(c_A, c_B))$. Integration of Eq (9) is performed over an area where growth rate is above a chosen threshold. Throughout the article this threshold is set to $y = 0.2$. Positive or negative values of LI suggest antagonistic or synergistic interaction, respectively.

3 Numerical solutions

We evaluated the steady-state solution of the system Eqs (6) by forward time integration of the differential equations. We used $\lambda_0 = 2\ \mathrm{h}^{-1}$, which is the drug-free growth rate in rich lysogeny broth medium at 37°C [2]. We rescaled the time by defining $t = T\tau$, where $T = (\alpha_A + \alpha_B)^{-1} \times (1/\lambda_0)$ and τ is a real number. Similarly, we rescaled concentrations by defining a reference concentration as $M = \left(1/\mathrm{IC}^*_{50,A} + 1/\mathrm{IC}^*_{50,B}\right)^{-1}$; concentrations of all species were rescaled by M. To automatically scale the antibiotic concentrations axes in units of IC_{50}, we set $\mathrm{IC}^*_{50} = 2\alpha/\left(\alpha^2 + 1\right)$. To mimic the situation in which exponentially growing unperturbed bacterial culture is exposed to antibiotic, we set the initial condition to $y = 1$; the rest of the species were set to 0. We integrated the rescaled differential equations forward in time until $\tau = 9 \times 10^3$ by using Mathematica function `NDSolve`. In computation, K_D and k_{on} values were set for both antibiotics to 0.1 μM and 100 $\mu\mathrm{M}^{-1}\mathrm{h}^{-1}$, respectively. The results are largely invariant of the choice of these numeric constants as long $k_{\mathrm{on}} \gg \kappa_t$ and K_D is roughly between 0.01−1 μM.

4 Analytical solution in the limit of strong inhibition and reversible binding

The system of ODEs [Eqs (6)] can be linearized for near-zero growth rates (*i.e.*, $\lambda \ll \lambda_0^*, \lambda_0$) which is the case when the external concentrations of antibiotics are high enough ($a_{\mathrm{ex},i} \gg \mathrm{IC}_{50,i}$). In the latter case one can postulate two constraints: (i) Ribosome synthesis is up-regulated to the theoretical maximum, *i.e.*, $r_{\mathrm{tot}} = r_{\mathrm{max}}$ and (ii) internal concentration of the i-th antibiotic is $a'_i \approx a_{\mathrm{ex},i} p_{\mathrm{in},i}/p_{\mathrm{out},i}$. These constraints eliminate the dynamical equations for internal concentrations of antibiotics [Eq (6a)]. The fully specified and expanded system of equations reads

$$\dot{r}_u = -(r_u - r_{\mathrm{min}})(k_{\mathrm{on},A} a'_A + k_{\mathrm{on},B} a'_B) + r_{b,A} k_{\mathrm{off},A} + r_d k_{\mathrm{off},B}, \tag{S4.10a}$$

$$\dot{r}_{b,A} = k_{\mathrm{on},A}(r_u - r_{\mathrm{min}}) a'_A + \delta_{\mathrm{off},B} k_{\mathrm{off},B} r_b^{AB} - \delta_{\mathrm{on},B} k_{\mathrm{on},B} r_{b,A} a'_B - k_{\mathrm{off},A} r_{b,A}, \tag{S4.10b}$$

$$\dot{r}_{b,B} = k_{\mathrm{on},B}(r_u - r_{\mathrm{min}}) a'_B + \delta_{\mathrm{off},A} k_{\mathrm{off},A} r_b^{AB} - \delta_{\mathrm{on},A} k_{\mathrm{on},A} r_d a'_A - k_{\mathrm{off},B} r_d, \tag{S4.10c}$$

$$\dot{r}_d = \delta_{\mathrm{on},A} k_{\mathrm{on},A} r_d a'_A + \delta_{\mathrm{on},B} k_{\mathrm{on},B} r_{b,A} a'_B - r_b^{AB} \left(\delta_{\mathrm{off},A} k_{\mathrm{off},A} + \delta_{\mathrm{off},B} k_{\mathrm{off},B}\right). \tag{S4.10d}$$

The system is linear and thus the steady-state solution exists in a closed and unique form. We initially derive the stationary solution for independent binding, *i.e.*, $\delta_{\sigma,i} = 1$:

$$r_u = \frac{\Delta r}{(1 + a'_A/K_{D,A})\,(1 + a'_B/K_{D,B})} + r_{\min}, \tag{S4.11a}$$

$$r_{b,A} = \Delta r \frac{a'_A}{a'_A + K_{D,A}} \frac{1}{1 + a'_B/K_{D,B}}, \tag{S4.11b}$$

$$r_{b,B} = \Delta r \frac{1}{1 + a_A/K_{D,A}} \frac{a'_B}{a'_B + K_{D,B}}, \tag{S4.11c}$$

$$r_d = \Delta r \frac{a'_A}{a'_A + K_{D,A}} \frac{a'_B}{a'_B + K_{D,B}}. \tag{S4.11d}$$

Each product term can be rewritten as a function of $a'_i/K_{D,i}$, which can be further transformed by noting that $K_{D,i}p_{\mathrm{out}_i} = (\lambda_0\alpha_i)^2/(4\kappa_t)$ and $a_{\mathrm{ex},i}p_{\mathrm{in},i} = c_i \times \left[\Delta r\lambda_0\left(\alpha_i^2+1\right)\right]/4$. Together, these expressions allow rewriting Eq (S4.11a) using variables $c'_i = c_i \times \left[(\alpha_i^2+1)\lambda_{\max}\right]/(\alpha_i^2\lambda_0)$, which finally leads to the Eq (10). To obtain Eq (11) the general solution of Eqs (S4.10) is evaluated; the latter is further simplified by assuming that $k_{\mathrm{off},A} \approx k_{\mathrm{off},B}$, which approximately holds for reversibly binding antibiotics.

5 Effect of dose-response curve concavity on the shape of isoboles

For intermediate values of $\alpha \sim 1$ and increasing δ, we observed that the isoboles of the dose-response surface at lower drug concentrations indicate strong antagonism, whereas at higher concentrations, they indicate synergism (Fig S1A). Is it possible to determine a concentration value above which increasing δ

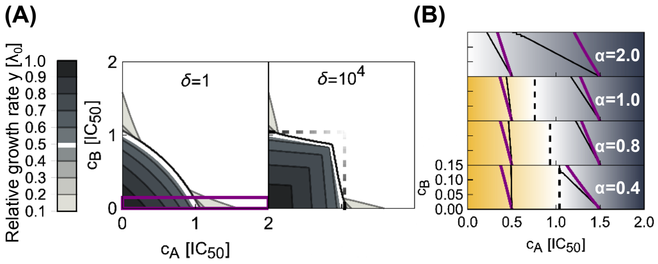

Figure S1: Dose-response surface can locally have both antagonistic and synergistic isoboles, depending on the concavity of the individual dose-response curves. (A) Examples of dose-response surfaces with $\alpha = 0.4$ and with either independent (left, $\delta = 1$) or strongly cooperative binding (right, $\delta = 10^4$). Purple box highlights the area showcased in (b). Dashed black lines denote an approximate area in which increasing δ increases antagonism. (B) A detail [highlighted rectangle in (A)] of dose-response surfaces for different values of α. For $\alpha < 2$ black dashed line denotes the boundary below which the increasing cooperativity δ increases antagonism (shades of yellow); above it synergistic character is enhanced (blue). Two isoboles are showcased for each example: purple and black lines correspond to $\delta = 1$ and $\delta = 10^4$, respectively. Note, that below c_{inf} isoboles in the case of strong cooperativity become steeper than the independent ones; above c_{inf} isoboles become shallower, which is indicative of synergy.

will cease to increase antagonism but rather increase synergism? Intuitively, the interaction-characteristic shape of isoboles is determined by the sign of the mean curvature, the latter being defined as

$$K_a = (k_1 + k_2)/2 \tag{S5.12}$$

where k_1 and k_2 are principal curvatures. Full dependency of K_a is not accessible as an analytical expression for $y(c_A, c_B)$ is not known; however in the limit $\delta \to \infty$ the antagonistic isoboles become nearly perpendicular to the axes, thus rendering all derivatives along perpendicular directions equal to zero. Hence, the expression for K_a is simplified into:

$$K_a\big|_{c_B \to 0} \approx \frac{\mathrm{d}^2 y/\mathrm{d}^2 c_A}{2\left[1 + (\mathrm{d}y/\mathrm{d}c_A)^2\right]}. \tag{S5.13}$$

As the denominator is always positive the sign of K_a depends only on the sign of the second derivative along the axis.

To find this point, we need to determine the inflection point of the relative growth rate y along either of the axes. Here, we need to solve $\mathrm{d}^2y/\mathrm{d}^2c = 0$ for α; since y is given only implicitly in Eq (5), the second implicit derivative is calculated. This leads to inflection point at relative growth rate $y_{\mathrm{inf}} = \sqrt[3]{\alpha^2/4}$ for $\alpha > \alpha_{\mathrm{crit}}$, at which second derivative vanishes. Using Eq (5) the inflection concentration is evaluated as:

$$c_{\mathrm{inf}} = \frac{-\alpha^2 + 2^{4/3}\alpha^{2/3}}{1+\alpha^2}. \tag{S5.14}$$

From the expression for y_{inf} we observe that we have a relevant solution only for $\alpha < 2$; this leads to the first conclusion–the intermediate regime of surfaces having both antagonistic and synergistic contours exists only up to $\alpha = 2$. This also implies that for every response parameter α, given a high enough concentration of an antibiotic, the isoboles will become synergistic. However, at low growth rates at high concentrations the approximation [Eq (13)] becomes relevant (Fig S1B). Numerically computed dose-response surfaces for $\delta = 1$ and $\delta = 10^4$ illustrate that indeed above the c_{inf} character of the isoboles is different and that the transition happens due to vanishing second derivative.

6 Experimental considerations

We verified the results on antibiotic induced starvation and constitutive expression of resistance genes by measuring the growth rate in two-dimensional gradients as described in Ref. [2]. Briefly, concentration gradients were constructed in the wells of microtiter plates; multiple such plates were cycled through the plate reader, which measured the luminescence as the proxy of the cell density. From the time traces of luminescence the growth rate of the bacterial culture in the individual well was determined by identifying an exponential region and fitting the line to the log-transformed luminescence values–precise methods and experimental setup description is in Ref. [2]. Bacterial growth medium used in all experiments was lysogeny broth (LB). Antibiotics solutions were prepared from powder stocks and dissolved in ethanol (CHL, MUP), water (STR) or directly in LB [TET and CHL (for CERG experiment)] and filter-sterilized. Growth rates were normalized with respect to the antibiotic-free growth rate λ_0 to obtain $y = \lambda/\lambda_0$; concentrations were normalized with respect to IC_{50}, which was determined from fitting a Hill function $1/\left[1 + (a_{\mathrm{ex},i}/\mathrm{IC}_{50,i})^n\right]$. To construct the double-resistant strain we followed the methods described in Refs. [2] and [3] with appropriate modifications. We have cloned *cat* gene into a plasmid with pKD13 background (as per Ref. [2]), in which resistance gene was driven by a synthetic promoter $\mathrm{P}_{\mathrm{LlacO-1}}$ [4]. Promoter was unregulated (constitutive) as the background strain HG105 (MG1655 Δ*lacIZYA*) [5] is devoid of the entire *lac* operon, including the *lac*-repressor. We amplified the *tetA* and *cat* from the strain MS004A [6] and plasmid pZA32 [4], respectively. We were unable to clone *tetA* into a plasmid; we therefore assembled the kanamycin cassette, $\mathrm{P}_{\mathrm{LlacO-1}}$ promoter, and *tetA* gene with a *rrnB* terminator *in vitro* using HiFi Assembly Mix (NEB), PCR-amplified the fragment, and integrated it into the *intS* locus. CAT-coding gene was integrated into *galK* locus. Both CERGs were integrated into the chromosome by lambda-red recombineering, selected on kanamycin, which was followed by the resolution of the kanamycin cassette by FLP-resolvase [7, 3, 8, 2, 9]. We verified modified chromosomal segments with PCR and Sanger sequencing.

7 Combinations of translation inhibitors with drugs that alter growth law parameters

We elaborated in the main text on a specific example of a starvation mimicking antibiotic, which invokes a response that is similar to shifting bacteria to poorer media (*i.e.*, change in λ_0). As growth laws capture this change, a response could be analytically predicted and was experimentally tested. Below we show two more hypothetical examples of antibiotic combinations in which translation inhibitor is combined with a drug that alters growth law parameters. We consider the case in which this hypothetical drug alters either r_{min} alone or in concert with r_{max}; in either case a shift in growth laws happens, which affects both dynamic range Δr as well as the apparent response parameter α. Here, we note that there is some experimental data illustrating that such shift could be achieved by antibiotics inhibiting *transcription*,

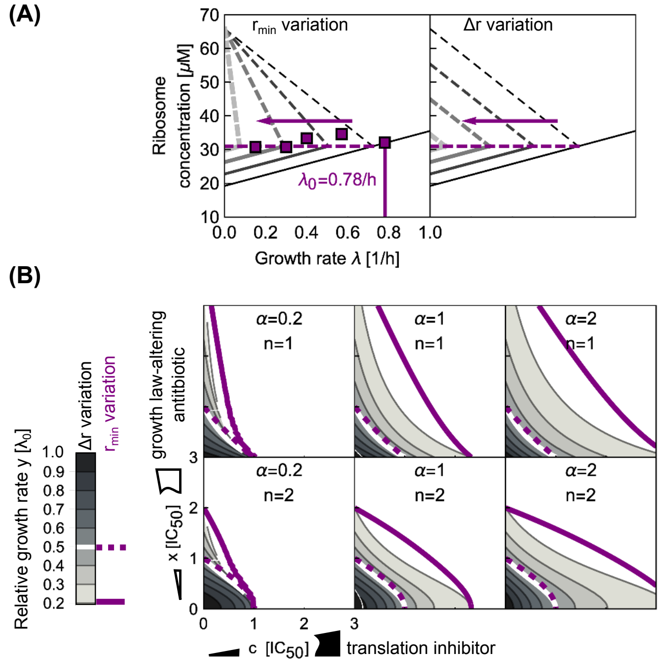

Figure S2: Impact of the variation in $r_{\rm min}$ and Δr on drug interactions. (A) Changes in growth laws as a function of variation in $r_{\rm min}$ (left) and Δr (right). Purple arrow denotes the effect of growth law-varying drug, which alter the "origin" of the translation inhibition growth law line (dashed gray lines). Slope of dashed lines is altered when $r_{\rm min}$ is varied; when $r_{\rm max}$ is varied simultaneously with $r_{\rm min}$ the slope is invariant of the "origin." Purple squares is data describing the ribosome abundance in response to rifampicin from Ref. [10]; we converted reported RNA/protein measurements to ribosome concentrations by fixing the drug-free data point to the ribosome concentration $r_u \approx 32.0\ \mu$M as determined by the Eq (1) at $\lambda_0 = 0.78\ {\rm h}^{-1}$. Note, that the data points lie approximately on the horizontal dashed line. (B) Examples of dose-response surfaces for different translation inhibitors (concentration c; different response parameters α) and different dose-response curves for Δr- and $r_{\rm min}$-varying antibiotic (concentration x; Hill-function with different steepness parameter n). For easier comparison we overlaid the dose-response surfaces; the purple contours correspond to Δr-varying case.

e.g., rifampicin (Fig S2A and supplementary information of Ref. [10]). However, the combined effect of transcription and translation inhibition of translation machinery has not be experimentally assessed.

If we consider an antibiotic that affects the $r_{\rm min}$, yet it does not affect the κ_t (Fig S2A), then the following modification to the growth law arises:

$$\begin{aligned} \lambda_0' = \lambda_0 g_{\rm min}(x) &= \kappa_t \left(\frac{\lambda_0}{\kappa_t} + r_{\rm min} - r_{\rm min}' \right) \\ \implies r_{\rm min}' &= r_{\rm min} + \frac{\lambda_0 \left[1 - g_{\rm min}(x)\right]}{\kappa_t}. \end{aligned} \tag{S7.15}$$

Here, we denoted by $g_{\rm min}(x)$ a dose-response function of a $r_{\rm min}$-varying antibiotic and x its concentration; $r_{\rm min}'$ denotes an apparent new $r_{\rm min}$ as a function of $g_{\rm min}(x)$. Thus, the reduced dynamic range reads $\Delta r' = r_{\rm max} - r_{\rm min}' = \Delta r - [1 - g_{\rm min}(x)]\, \lambda_0/\kappa_t = \Delta r \left\{1 - [1 - g_{\rm min}(x)]\, r_0/\Delta r\right\}$, where we defined $r_0 = \lambda_0/\kappa_t$. Taken together, these relations rescale the response parameter $\alpha = \alpha_F/g_{\rm min}(x)$ (where α_F is the response parameter for $x = 0$), while the apparent concentration becomes $c' = c/\psi_{\rm min}$, where

$$\begin{aligned} \psi_{\rm min} &= \frac{\mathrm{IC}_{50}}{\mathrm{IC}_{50,F}} = \\ &= \frac{\alpha_F^2 + g_{\rm min}^2(x)}{g_{\rm min}(x)\,(\alpha_F^2 + 1)} \times \left\{ 1 - \frac{[1 - g_{\rm min}(x)]\, r_0}{\Delta r} \right\}. \end{aligned} \tag{S7.16}$$

If we consider that $g_{\min}(x) = 1/(1 + x^n)$, where x is the concentration of $r_{\min}$-varying antibiotic measured in units of IC_{50}, we can calculate the whole dose-response surface as

$$y(c, x) = g_{\min}(x) \times f\left(\alpha_F / g_{\min}(x), c/\psi_{\min}\right), \tag{S7.17}$$

where f is a translation inhibitor dose-response curve. Examples are shown in Fig S2B.

Model variation discussed above can be expanded in a model in which both $r_{\min}$ and $r_{\max}$ are varied simultaneously (Fig S2A). Here, we assume that $r_{\min}$ increases and $r_{\max}$ decreases in concert such that they have the same value at $g_{\Delta\mathrm{r}} = 0$ (Fig S2A). It follows

$$\begin{aligned} \Delta r' &= r'_{\max} - r'_{\min} = \\ &= \underbrace{r_{\max} g_{\Delta r}(x) + [1 - g_{\Delta r}(x)]\,(r_0 + r_{\min})}_{r'_{\max}} - \\ &- \underbrace{\{r_{\min} + [1 - g_{\Delta\mathrm{r}}(x)] r_0\}}_{r'_{\min}} = \Delta r g_{\Delta r}(x). \end{aligned} \tag{S7.18}$$

The response parameter α is rescaled as above; the ratio $\psi = \mathrm{IC}_{50}/\mathrm{IC}_{50,F}$ takes a slightly different form:

$$\psi_{\Delta r} = \frac{\alpha_F^2 + g_{\Delta r}^2(x)}{g_{\Delta r}(x)\,(\alpha_F^2 + 1)} \times g_{\Delta r}(x). \tag{S7.19}$$

With this at hand, dose-response surfaces can be evaluated (Fig S2B). Here we note that the both models give the same result if $r_0 = \Delta r$, which requires $\lambda_0 = \Delta r \kappa_t$. This is intuitive since the ribosome concentration is already at its maximum and only $r_{\min}$ can be varied.

References

1. Greulich P, Scott M, Evans MR, Allen RJ. Growth-dependent bacterial susceptibility to ribosome-targeting antibiotics. Mol Syst Biol. 2015;11:796–807.

2. Kavčič B, Tkačik G, Bollenbach T. Mechanisms of drug interactions between translation-inhibiting antibiotics. Nat Commun. 2019;11(4013):1–14.

3. Deris JB, Kim M, Zhang Z, Okano H, Hermsen R, Groisman A, et al. The Innate Growth Bistability and Fitness Landscapes of Antibiotic-Resistant Bacteria. Science. 2013;342:1237435.

4. Lutz R, Bujard H. Independent and tight regulation of transcriptional units in *Escherichia coli* via the LacR/O, the TetR/O and AraC/I_1-I_2 regulatory elements. Nucleic Acids Res. 1997;25(6):1203.

5. Garcia HG, Lee HJ, Boedicker JQ, Phillips R. Comparison and Calibration of Different Reporters for Quantitative Analysis of Gene Expression. Biophys J. 2011;101:535.

6. Steinrück M, Guet C. Complex chromosomal neighborhood effects determine the adaptive potential of a gene under selection. eLife. 2017;6:e25100.

7. Datta S, Constantino N, Court DL. A set of recombineering plasmids for gram-negative bacteria. Gene. 2006;379:109.

8. Datsenko KA, Wanner BL. One-step inactivation of chromosomal genes in *Escherichia coli K-12* using PCR products. Proc Nat Acad Sci USA. 2000;96(12):6640.

9. Cherepanov PP, Wackernagel W. Gene disruption in *Escherichia coli*: Tc^R and Km^R cassettes with the option of Flp-catalyzed excision of the antibiotic-resistance determinant. Gene. 1995;158:9.

10. Scott M, Gunderson CW, Mateescu EM, Zhang Z, Hwa T. Interdependence of Cell Growth and Gene Expression: Origins and Consequences. Science. 2010;330:1099.
