# Supplementary material for: Minimal biophysical model of combined antibiotic action
Source: PLoS Comput Biol. 2021 Jan 7;17(1):e1008529. doi: 10.1371/journal.pcbi.1008529 (PMC7817058; doi:10.1371/journal.pcbi.1008529)
Supplement: Attachment — Submitted filename: point-by-point_response.pdf [file pcbi.1008529.s002.pdf]

Reviewer #1:

This is an interesting and significant manuscript which presents and analyses a minimal model for the combined action of pairs of ribosome targeting antibiotics on a bacterial cell. Understanding the combined action of antibiotics is crucially important since many clinical therapies involve combinations of antibiotics, and it is well known that interactions can be either synergistic or antagonistic, or even suppressive. However, there are few quantitative predictive models for drug interactions (or even for the action of individual antibiotics). Therefore this work makes an important contribution to the field. The work presented is careful, thorough, mostly clearly described, and supported by some original experimental data. I support its publication in PLoS Computational Biology, although I have some suggestions for the authors to consider.

We appreciate the reviewer's thorough assessment of our work, the positive comments, and the helpful constructive suggestions. We revised the manuscript accordingly and added several additional explanations of our results.

Suggestions:

1. The caption of Fig 2 should cite Scott et al (2010) [9] and Greulich et al (2015) [10].

This has been corrected.

2. It would be useful to give the expression for the bound ribosome concentration rb before Eq (2), since that is needed in Eq (7).

We have added a definition of $r_b$ right after Eq. (2) [lines 100-101] and spelled out the definition of $r_b$ before Eq. (7) [lines 171-173].

3. On line 94, ref [10] is not appropriate (just ref. [9])

We agree that the relation in Eq. (2) was first reported in ref. [9] [line 102]. We use the notation from ref. [10] for this result but we agree that this citation can be removed here since we cite this work elsewhere.

4. There are blank spaces in place of section numbers in several places, eg before Eq 6, line 305, line 480.

This has been corrected.

5. Lines 151-153 state that "in general the parameters delta can vary continuously to capture any change in ribosome binding of one antibiotic due to binding of the second" – I don't think this is quite true. For example if the binding becomes non-linear this would not be captured?

We agree with the reviewer on this point. We added an additional requirement to this explanation: "*... vary continuously to capture any changes in ribosome binding of one antibiotic due to the binding of the second, as long as the binding is described by mass-action kinetics*." [lines 161-164]

6. In lines 159-163 the wording is confusing where it refers to "bound to one antibiotic" and "single-bound antibiotic" – here we are anyway only talking about the case of single binding?

7. Before Eq (7) the parameter xi appears with no explanation, and in Eq (7) the expression for rb = Delta r(1-lambda/lambda0) appears without explanation – this should have been explained explicitly before Eq (2). Also it should be made clear that the final relation applies only along the isobole.

8. In Eq (8) it should be made clear this in the steady state. Also after Eq (8) it was not clear to me what is meant by the proportionality constant.

9. Eq (8) is not actually an equation for the isobole (that would be an equation that links the two antibiotic concentrations for fixed growth rate). I think the statement "Eq (8) corresponds to a linear isobole" would be clearer if an actual equation for the isobole can be stated.
10. Lines 171-173 seemed a little out of place and perhaps distracting.
We thank the reviewer for raising these concerns (6.-10.) and suggesting ways of alleviating them. We have rewritten these parts and included additional derivations to make them clearer. [lines 165-190]

11. Lines 187-188 a sentence or two to describe Fig 3c in more detail might be useful for clarity.
We added an additional interpretation of the phase diagram into the main text. [lines 204-210]

12. Lines 189-196: the wording here implies a time-line (antibiotic 1 binds before antibiotic 2) that is not accurate: the model does not specify which antibiotic binds first. The explanation here could be rewritten to reflect this.
We agree that the current wording can be misleading. We have reworded this part accordingly. [lines 211-219]

13. Lines 203-204 the wording is a little hard to follow, e.g. "growth is inhibited close to zero" – what is the assumption here, that the antibiotic concentration is low so there is little inhibition?
We thank the reviewer for pointing out a potential misunderstanding: here, we meant that the growth rate is very low, almost zero. Therefore, the total ribosome concentration is close to $r_{max}$ as formalized by Eq. (2). We rephrased this sentence to make this point clearer. [lines 222-223]

14. In the caption of Fig 4b, it states that the dashed line is for antibiotics with different alpha values. But the axis label seems to imply the alpha values are the same.
Indeed this labeling was confusing. For the solid line, the values of $\alpha_A$ and $\alpha_B$ are identical, while for the dashed line the value of $\alpha_A$ varies but that of $\alpha_B$ is fixed. We added new labels to the lines in this plot and changed the axis label to clarify this issue.

15. Lines 225-230 the statement "it is unclear if mutual stabilization of binding necessarily leads to synergy" is not backed up by the text that follows. Actually it seems to imply that mutual stabilization of binding does lead to synergy; in contrast, it is if the two antibiotics mutually destabilize that the interaction is no longer synergistic.
We removed this part.

16. Lines 324-326 – this statement that the model can be generalized to other modes of antibiotic action is not clear to me. The model is based around the growth rate-dependent regulation of ribosome abundance. It is not obvious to me that it can easily be generalized to other modes of action. I feel that this statement is interesting but deserves a more in-depth justification, for example in the discussion section.
17. Lines 329-330 The statement that the growth rate is invariant of the target details as it can be recovered from first order kinetics is unclear to me. What is meant by first order kinetics here? In my view the core of the model is specific to ribosome-binding antibiotics.
We thank the reviewer for pointing out unclear parts of this segment (16.-17.). We expanded this part and added a new Figure 7 to make it clearer. [lines 338-370]

18. Line 377 onwards: it seems important to clarify here that the experiments were done with E. coli (rather than S. aureus which is mentioned early in the paragraph).
We added this clarification in the section on SMA [line 423] and later in the section on CERG [line 480].

19. Lines 396-7 “recent work” – it is perhaps not that recent (2013).
We reworded this sentence. [lines 443-444]

20. Line 428 The word “drastic” here is perhaps overkill since the previous sentence states that the effect is “slight”.
We agree and deleted “drastic” here. [lines 475-476]

21. The result that the presence of resistance genes can qualitatively change the nature of the drug interaction is very nice and could perhaps be made more prominent in the abstract (it is there but is rather buried under other things).
We modified the abstract to describe the extensions of the model in more detail.

22. I also wonder whether how this phenomenon (effect of resistance genes) depends on the growth rate?
We thank the reviewer for raising this interesting point. According to the theoretical framework by Scott et al., the expression of a constitutively expressed gene should be close to zero when the drug-free growth rate $\lambda_0$ approaches the maximal attainable one ($\lambda_{max} = \Delta r \times \kappa_t \approx 2.8\, h^{-1}$). Thus, at rapid growth the strain should behave as a sensitive WT strain. Yet, at slower growth, expression increases, resulting in a more pronounced effect of the resistance genes. We discuss this in more detail [lines 451-462; 502-506]; furthermore, we have added a new panel (a) in Figure 9 illustrating these considerations.

Reviewer #2:

The manuscript studies a mathematical model for the combined action of two or more growth-inhibiting antibiotics. In these combinations at least one of the studied antibiotics is a translation-inhibiting antibiotic therefore the authors base their model on a previous model (Greulich et al, 2015) for the mode of action of a translation-inhibiting antibiotic, which takes into account the coupling between antibiotic action and bacterial growth.

The authors predict the interaction of antibiotics (antagonistic, independent, or synergistic) for different combinations of antibiotic types. In particular, for antibiotics which simultaneously bind to the ribosome, they study how binding interactions between antibiotics on the same ribosome (e.g. of allosteric origin) affect the combined drug efficacy. In that scenario, the combined effect is explicitly modelled as an extension of the model (Greulich et al. 2015), and it is predicted that the combined drug effects -- antagonistic or synergistic -- are enhanced if drug molecule binding is synergistic, and weakened if drug molecule binding is antagonistic. This is surprising when considering the naive view that synergistic drug binding should generally lead to a synergistic drug effect. This is an interesting prediction worth to be tested experimentally.

It is further studied how translation-inhibiting antibiotics interact with starvation-mimicking antibiotics and how the presence of resistance genes affects the drug interaction. Those predictions are also tested experimentally in bacterial growth assays where antibiotics are applied and genetically modified bacterial strains are used to study the effect of resistance genes.

This study is a very interesting and solid piece of work, with a thorough, comprehensive study of a realistic model (partially confirmed in the past) and experimentally testable predictions. Some of these

predictions are directly tested experimentally in this work. Predictions about the effect and interaction of antibiotics (e.g. dose-response curves) as done in this work are of high medical value.

The only concern I have is that the authors decided to test some of their predictions experimentally, but others not. While it is generally a plus of this study that model predictions are experimentally tested, there is the risk of a confirmation- or selective bias if only some predictions are experimentally tested. It is therefore essential that the authors give a clear rationale why other predictions have not been tested experimentally, and argue thoroughly that no selection bias occurs. Of course, if possible, doing the corresponding experiments for those predictions would be preferable and strengthen this work even further.

We thank the reviewer for her/his careful assessment of our work, the positive comments, and the constructive suggestions for improvements. The reviewer is correct that we tested only some of our theoretical findings experimentally. This was done for practical reasons:

(i) We presented the correspondence between experiments and the simplest variants of the model (independent and competitive binding) in another, more experimentally-focused article [Kavčič, Tkačik & Bollenbach, Nat. Commun. 11 (2020), Ref. 12, previously cited as a preprint]. [lines 239-242]

(ii) We could not experimentally verify direct interactions between antibiotics (*e.g.,* for the pairs quinupristin-dalfopristin or lankamycin-lankacidin) because these drugs are either not commercially available or prohibitively expensive for our scale of experimentation. Additionally, a direct prediction of the dose-response surface requires the value for the parameter $\delta$, which can only be extracted from dedicated biochemistry measurements. [lines 274-275]

(iii) We constructed another CERG strain, bearing resistance to erythromycin (ERM) to observe the effect of CERGs on synergy between TET and ERM. However, the ERM dose required to completely inhibit the growth of this resistant strain was above the solubility limit of the drug. We considered solving this issue by (1) altering the permeability of the membrane or (2) by reducing the expression of the resistance gene genetically. In either case, it would be difficult to interpret the results. Additionally, ERM resistance protects ribosomes by modifying the ribosomal RNA and therefore causes a partitioning of ribosomes into susceptible and resistant subpopulations: this would require another modification of the model, which would obfuscate the interpretation. [lines 494-508]

In the present work, we experimentally verified only the most surprising and testable predictions of the model that we had not reported before. In sum, the experimental verification presented in this manuscript is limited by a combination of restricted commercial availability of reagents, genetic challenges, modeling considerations, and an overlap with previous results.

Further minor comments:

1. line 105: For completeness, it should be said that the form if s(lambda) stated here is only valid in the steady state (the steady state is only mentioned in the following sentence).
We added this clarification.

2. below Eq. 6, index are sometimes named "A" and "B" and sometimes "1" and "2". It would be good to be consistent in the notation here.

We changed the notation to avoid superscript indices and this intermingled designation of different antibiotics by letters and numbers, which was indeed confusing.

3. line 199: it is stated that large alpha means that binding is more reversible, however, it could also mean that antibiotic outflux of the cell is large.

This is correct. We rephrased the corresponding paragraph to clarify this point. [lines 220-222]

4. The authors prefer using Loewe's definition of drug interactions (antagonistic/independent/synergistic). Please argue why this definition is preferred, since in the following it is shown that this crucial classification depends sensitively on this definition. In particular, for the limit studied in Eq. (10), the interaction is synergistic in Loewe's view, but independent in Bliss' view, so the terms "synergistic" and "independent" are just semantics at this point.

We agree that the choice of the drug interaction definition is largely arbitrary. Here, we decided to use the Loewe definition of additivity as a base expectation because it has a distinct advantage over Bliss independence: it can recognize if combined drugs act as substitutes for one another. This is a particularly important benchmark for the model: we *a priori* know the type of interaction for a combination of a drug with itself, which serves as a consistency check [lines 32-43]. The reviewer is correct in pointing out this subtle distinction on which our analysis is based: we added the point above to the main text and highlighted this distinction again in the text [lines 288-289].

5. line 323: It is stated that Eq. 16 is consistent with Eq. 14. This does not become clear at this point. Could the authors show this at this point or refer to an Appendix?

We expanded this section to make the derivation and the correspondence between both simplified-mechanistic and mechanism-independent models clearer. [lines 338-370]

6. In Fig. 7c there are substantial deviations between experimental data and model predictions for streptomycin. Please comment on this deviation.

We thank the reviewer for this remark. We revisited the data analysis for this part and now show the experimental data with error bars to depict the inaccuracies in the determination of relative changes in $IC_{50}$. We also included the functional bounds of the $\Psi(g(c_s),\alpha)$ [Eq. (18)] dependency [ $1/g(c_s)$ and $g(c_s)$ for $\alpha \to \infty$ and $\alpha \to 0$, lines 408-409] to show that the data are within the limits of the model. Since the STR data lie on the bound of the model [ $g(c_s)$, corresponding to low $\alpha$ ], we attribute the deviations to a poor fit of the response parameter. Fitting the response parameter to steep dose-response curves (such as the one of STR) is challenging. When $\alpha$ is below $\alpha_{crit}$, the part of the dose-response curve corresponding to high growth rates ( $c < 1$, where data is plentiful) changes little, while the part corresponding to very low growth rates ( $c > 1$, where data is scarce) changes significantly. This issue is also apparent in the paper by Greulich *et al.*, where the model fails to faithfully describe the steep dose-response curves (see *e.g.*, Fig. 3A,B in Greulich *et al.*). We now explain this issue in the main text [lines 428-433]. We note, however, that the main point here is the qualitative difference in the dependency of the relative $IC_{50}$ on SMA inhibition (decreasing versus monotonically increasing for dose-response curves with $\alpha < 1$ and $\alpha > 1$, respectively).

7. At several points in the text, references to Appendix 5 are made for experimental details, but these are actually given in Appendix 6.

We corrected the referencing.
